# Genetic Deletion of a Single Immunodominant T-cell Response Confers Susceptibility to Virus-induced Demyelination

**DOI:** 10.1111/j.1750-3639.2007.00062.x

**Published:** 2007-04-01

**Authors:** Kevin D Pavelko, Larry R Pease, Chella S David, Moses Rodriguez

**Affiliations:** 1 Department of Immunology, Mayo Clinic College of Medicine Rochester, Minn; 2 Department of Neurology, Mayo Clinic College of Medicine Rochester, Minn

## Abstract

An important question in neuropathology involves determining the antigens that are targeted during demyelinating disease. Viral infection of the central nervous system (CNS) leads to T-cell responses that can be protective as well as pathogenic. In the Theiler’s murine encephalomyelitis virus (TMEV) model of demyelination it is known that the immune response to the viral capsid protein 2 (VP2) is critical for disease pathogenesis. This study shows that expressing the whole viral capsid VP2 or the minimal CD8-specific peptide VP2_121-130_ as “self” leads to a loss of VP2-specific immune responses. Loss of responsiveness is caused by T cell-specific tolerance, as VP2-specific antibodies are generated in response to infection. More importantly, these mice lose the CD8 T-cell response to the immunodominant peptide VP2_121-130_, which is critical for the development of demyelinating disease. The transgenic mice fail to clear the infection and develop chronic demyelinating disease in the spinal cord white matter. These findings demonstrate that T-cell responses can be removed by transgenic expression and that lack of responsiveness alters viral clearance and CNS pathology. This model will be important for understanding the mechanisms involved in antigen-specific T-cell deletion and the contribution of this response to CNS pathology.

## INTRODUCTION

Immunologic tolerance has been defined in many ways and can be acquired through several different mechanisms. T-cells that encounter peripheral antigen in the absence of co-stimulation are either rendered anergic to that particular antigen or develop into T regulatory cells (41). During thymic development T cells are selected for their recognition of self antigens in the context of major histocompatibility complex (MHC) molecules where a majority of high affinity clones are then deleted from the population of thymic emigrants (10). Recent evidence has suggested that a portion of these high affinity clones may assume an alternative pathway that results in the development of antigen-specific T regulatory cells (1). From the perspective of autoimmune disease however, the goal of tolerance induction is to abrogate a specific T-cell response that is initiating or perpetuating an immune-mediated pathology, while leaving the remainder of the T-cell repertoire intact. The manipulation of antigen-specific T cells is being used as therapy for a number of autoimmune diseases, including diabetes, myasthenia gravis and multiple sclerosis (MS) (19, 34). These therapies have been used to modulate T cell receptor/MHC/peptide interactions in both a peptide-specific and a peptide-non-specific manner in the hopes of inducing non-responsiveness to the target antigen. The precise mechanisms that lead to the therapeutic effect, however, have not been identified. Several hypotheses have been proposed, including deletion, anergy or the induction of T regulatory cells (8, 39). A consensus has not been reached and further study into the mechanism of tolerance induction is needed to verify the optimal treatment strategy.

Our laboratory uses the Theiler’s murine encephalomyelitis virus (TMEV) model of multiple sclerosis to study the mechanisms involved in immune-mediated demyelination. Intracranial infection with TMEV leads to an encephalitis that is resolved in all strains of mice, however, certain strains develop a chronic infection and demyelination in the spinal cord white matter (30). The MHC has been shown to be critical for TMEV-induced immunopathology, particularly the H-2D region of the class I locus (29). H-2D^b^ mice resolve the encephalitis associated with TMEV infection and generate a robust CD8+ T-cell response that leads to viral clearance (20). In contrast, mice of the H-2^f,m,s,q,u^ haplotypes resolve the encephalitis associated with infection but fail to clear the virus and develop a chronic infection in the spinal cord that is associated with axonal demyelination in the spinal cord white matter (30).

The viral capsid protein viral protein 2 (VP2) has been shown to be targeted by the immune system during TMEV infection. These responses include B-cell responses as observed by VP2-specific antibody (4), CD4 T-cell responses which secrete IFN-γ (14) as well as CD8 T-cell responses that have cytolytic activity (3). One peptide antigen from VP2, however, has been shown to be critical for resistance to TMEV infection. The peptide VP2_121-130_ (FHAGSLLVFM) of TMEV is an immunodominant peptide recognized by CD8+ T cells in the context of H-2D^b^(12) and its recognition is essential for the protection from viral persistence. Further, depletion of antigen-specific CD8+ T cells before infection using VP2_121-130_ peptide blocked the resistance to TMEV-induced demyelination, demonstrating the importance of this antigen for viral clearance and susceptibility to demyelination (20).

To test whether we could delete antigen-specific T cells using a genetic approach we generated mice that express the whole VP2 capsid or the VP2_121-130_ peptide as self antigens. We chose the whole VP2 protein as well as the antigenic peptide so that we could rule out the possibility that sequences flanking the VP2_121-130_ peptide would be necessary for presentation on MHC class I molecules in the thymus. Our hypothesis was that expression of these transgenes in mice would lead to tolerization of T cells reactive to these antigens and that this would lead to a change in neuropathology.

This model allows us to monitor the induction of tolerance by assessing the presence of pathology not usually seen in resistant mice. Although previous studies have been able to demonstrate depletion of antigen-specific T cells using peptides (5), evidence has also emerged that peptide therapy may not be effective at deleting T cells (28, 38). In addition, peptide therapy is being used as a method to promote antigen-specific T-cell activation and immunity (6) and this therapy in an inflammatory setting could potentially generate an adverse immune response (2, 13, 15, 27). Therefore, it is important to understand the mechanisms involved in the deletion of an antigen-specific response to self antigen expressed in the thymus, especially when considering what is known about the limitations and heterogeneity of peptide therapy.

This study shows that transgenic expression of the capsid protein VP2 and the immunodominant peptide VP2_121-130_ led to deletion of VP2 and VP2_121-130_ specific CD8+ T cells from the inflammatory infiltrate isolated from the brains of infected mice. We demonstrate that deletion of these epitope-specific T cells had a dramatic effect on the ability of resistant mice to clear virus from the central nervous system (CNS) and show that this results in the development of demyelination in the spinal cord white matter. Further, we show that while antibody responses to TMEV during infection were not changed in VP2 transgenic mice, these mice were unable to generate VP2-specific antibodies when immunized with recombinant VP2 protein, demonstrating a potential defect in CD4 T helper cell responses or B cell responses. Our findings are the first to demonstrate an epitope-specific transgenic deletion of CD8+ T cells that influences pathologic sequelae. These findings will be important in trying to better understand the mechanisms underlying the deletion of antigen-specific T cells.

## MATERIALS AND METHODS

### Virus and infection

The Daniels strain of TMEV was used for all experiments. Mice were injected intracranially with 2 × 10^5^ plaque forming units of TMEV in a volume of 10 µL. Analyses were performed on day 7 and day 45 post TMEV inoculation.

### Mice

All non-transgenic FVB mice were obtained from Jackson Laboratory (Bar Harbor, ME). FVB D^b^ mice were provided by Dr Larry Pease (20).

### Generation of transgenic mice

Transgenic mice were generated by cloning TMEV cDNA into the eukaryotic expression vector pUB6 which contains an upstream human ubiquitin c promoter (Invitrogen, Carlsbad, CA). Complementary DNAs were directionally cloned from the TMEV clone pDAFL3 (24) using a BamHI site on the 5′ end of the cloned fragment and an EcoRV site on the 3′ end. All constructs were cloned while maintaining the HisTag included in the vector, thereby allowing the possibility of identifying these genes by using this marker. The VP2_121-130_ construct was cut with Bgl II and Nsp I to release an expression fragment of length 1567 base pairs which contained minimal vector sequence. This construct encoded a 33 amino acid fragment that included the 10 amino acids comprising the immunodominant peptide plus 5 amino acids on the carboxyl and amino terminal ends to exclude the possibility that flanking amino acids may be necessary for loading the fragment onto class I molecules in the endoplasmic reticulum. In addition, the start codon in the context of an appropriate Kozak sequence were added on the 5′ end, which gave an additional methionine and aspartic acid residue on the amino terminus of the construct. Further, an additional 11 amino acids were added on the carboxy terminus, which included a 5 amino acid linker attached to the 6XHisTag. VP2 and 3D constructs were cut with Bgl II and Pvu II to yield fragments of 2352 bp and 2935 bp. The VP2 construct encoded a 279 amino acid product which included the 267 amino acids of VP2. The 3D polymerase construct encoded a fragment that included all 462 amino acids of this viral RNA polymerase. All fragments were gel purified and sequenced before injection into embryos. Gel purified cDNA was injected into FVB embryos for implantation into pseudo-pregnant females. All embryo injections and implantations were done at the Mayo Clinic Transgenic Core Facility under the direction of Dr Chella David. Tail samples from the offspring were used to obtain genomic DNA for determination of transgene integration. Forty-nine potential founder mice were screened by polymerase chain reaction (PCR) for integration of the VP2 transgene. Two mice positive for the transgene were used to establish 2 VP2 transgenic lines. Forty-six potential founder mice were screened for the integration of the VP2_121-130_ transgenic construct. Three mice were determined to be positive for transgene integration by PCR, two lines of transgenic mice were established with mice having the highest integration as determined by semi-quantitative PCR. Thirty-nine potential founder mice containing the 3Dpol were screened for transgene integration, seven mice were positive and two of these were used to establish lines. DNA samples were screened using primers for the particular TMEV gene as well as the ubiquitin c promoter region. All mice used in every experiment were screened by PCR prior to their use in subsequent assays. TMEV transgenic mice were then crossed to FVB D^b^ transgenic mice to generate a line of TMEV resistant transgenic mice that express H-2D^b^. All of these transgenic lines behaved similarly to the wild-type controls and no gross clinical or morphologic abnormalities were observed in the transgenic lines.

Expression of transgenes was assessed by RT-PCR and western blotting. Total RNA was isolated from the brain, spinal cord and thymus of transgenic mice to determine expression of the transgenes. Briefly, tissues were homogenized with a dounce homogenizer and samples of the homogenate were used to isolate RNA using the RNeasy kit (Qiagen, Valencia, CA, USA). Real time reverse transcription PCR was used to determine expression of transgenes as well as relative amount of transcript. Real-time RT-PCR was performed on the Lightcycler 2.0 (Roche Diagnostics, Indianapolis, IN, USA), using the Roche FastStart DNA master SYBR-Green PCR kit. Primers were designed to specifically recognize the individual transgenes as well as primer pairs that could recognize shared sequence between the transgenes. Crossing points were determined by SYBR green incorporation into the PCR product using the Lighcycler 3 software program (Roche Diagnostics, Indianapolis, IN, USA). Relative mRNA expression was determined by subtracting the crossing point of a particular sample from the crossing point determined in a mRNA negative sample, so that a lower crossing point reflects a higher relative mRNA expression. Negative values were possible because of the potential for non-specific priming caused by primer dimer formation. However, all gene products were verified by size on 1.5% agarose gels and the products were consistent with the relative mRNA expression determined by the crossing point data. In addition, samples were tested for the expression of glyseraldehyde-3-phosphate dehydrogenase to verify equal loading across samples.

Brain homogenates were subsequently run on SDS-PAGE and blotted onto nitrocellulose and expression of proteins was determined by blotting with an anti-HisTag antibody and rabbit anti-TMEV polyclonal antibody.

### Histology

After 7 or 45 days of infection with TMEV, mice were euthanized with an overdose of pentobarbital and perfused by cardiac puncture with either Trump’s fixative or 4% paraformaldehyde. Brain and spinal cord were removed and used for histologic and immunohistochemical assays. Spinal cords were serially sectioned, fixed with osmium tetroxide and every other section was embedded in araldite embedding compound (Polysciences Inc., Warrington, PA, USA). Two micron sections of 10–12 spinal cord pieces were cut and stained using paraphenylenediamine. Sections were analyzed for spinal cord pathology using a method previously described (26). The remaining spinal cord sections were embedded in paraffin and were used for subsequent immunohistochemical analysis.

Brains were removed, sectioned and assessed for pathologic severity as described previously (7). Briefly, two coronal cuts were made after removal from the skull, one through the optic chiasm and another through the infundibulum. The three sections were then embedded in paraffin for subsequent pathologic analysis and immunohistochemical staining. Hemotoxylin and eosin stained brain sections were used to assess the severity of brain pathology observed in 7-day infected animals. Pathology scores were assigned based on the degree of pathology in the following anatomic regions: cerebellum, brain stem, cortex, hippocampus, striatum and corpus collosum. Each area of the brain was graded on a scale of 0 to 4. The following criteria were used to assign scores for each region: 0 = no pathology; 1 = no tissue destruction with minimal inflammation; 2 = early tissue destruction and moderate inflammation; 3 = definite tissue destruction (parenchymal damage, cell death, neurophagia, neuronal vacuolation); 4 = necrosis (complete loss of tissue elements). Meningeal inflammation was assessed and graded as follows: 0 = no inflammation; 1 = one cell layer of inflammation; 2 = two cell layers of inflammation; 3 = three cell layers of inflammation; 4 = four or more cell layers of inflammation. All slides were photographed using an Olympus DP70 camera attached to an Olympus AX70 research microscope (Olympus America Inc., Center Valley, PA, USA).

### Generation of recombinant proteins

VP2 proteins were generated as has been described previously (16). Briefly, VP2 cDNA was cloned into the pET30 vector (EMD Biosciences, Inc., San Diego, CA, USA). The construct coded a protein fragment that included the complete 267 amino acids of VP2 plus an additional 66 amino acid which included an enterokinase cleavage site and a HisTag for purification. Plasmids were transformed into the BL21 (DE3) (EMD Biosciences, Inc., San Diego, CA, USA) strain of *Escherichia coli* for expression and were induced with isopropyl-beta-D-thiogalactopyranoside after cells reached the linear phase of proliferation. Cells were pelleted and washed with phosphate-buffered saline (PBS) before disruption by sonication. Cell lysates were used to immunize mice for VP2-specific responses. Proteins were further purified over a HisTag column for *in vitro* use. This was performed under denaturing conditions using 6M urea to solubilize inclusion bodies formed after induction of plasmids. Proteins used for *in vitro* assays were dialyzed into PBS before use.

VP1 and VP2 protein fragments were generated using the rapid translation system 100 *E. coli* HY Kit (Roche), which allowed us to generate cell-free fragments of VP1 and VP2. Overlapping cDNA constructs corresponding to TMEV amino acids VP1_92-190_, VP1_180-274_, VP2_1-100_, VP2_90-180_ and VP2_171-267_ were generated. cDNAs were cloned into the pIVEX2.4 vector (Roche Diagnostics Corporation, Indianapolis, IN, USA). Proteins were generated according to the manufacturer’s protocol. Briefly, 0.5 µg of vector cDNA was added to 12 µL of *E. coli* lysate along with 5X reaction mix, amino acids and reconstitution buffer, reaction was brought up to a final volume of 50 µL with water. Translation reaction mixtures were incubated at 30°C for 6 h, samples were stored at −20°C until they were used in protein blots.

### Isolation of central nervous system infiltrating lymphocytes and flow cytometric analysis

The following fluorescent conjugated antibodies were used to characterize T cell infiltrates from TMEV-infected mice: APC-conjugated anti-CD45 (Clone 30-F11), FITC-conjugated anti-mouse CD8a (Clone 53-6.7) and PE-conjugated anti-mouse CD4 (Clone H129.19).

H-2D^b^ tetramers were made by the National Institute of Allergy and Infectious Disease MHC Tetramer Core Facility. The peptide FHAGSLLVFM was used to identify T cells recognizing this peptide in the context of H-2D^b^. The human papillomavirus peptide E7 (RAHYNIVTF) which also binds to H-2D^b^ was used as a negative control. All peptides were generated at the Mayo Proteomics Research Center Peptide Synthesis Facility.

Lymphocytes were isolated as described previously (17). Briefly, 7-day infected mice were killed using isofluorane overdose, brain and spinal cords were removed aseptically and homogenized in 20 mL of RPMI media. Ninety percent Percoll/PBS was added to 30 mL and the sample was centrifuged at 27 000 g for 30 minutes at 4°C. The mononuclear cell band was removed and resuspended in ACK buffer to lyse red blood cells. Cells were counted, washed in RPMI and split into two fractions. Both fractions were analyzed using anti-CD45, anti-CD8 and anti-CD4 antibodies. One fraction was analyzed with a mouse D^b^ tetramer to the peptide VP2_121-130_ and the other to a control peptide E7.

### Immunohistochemistry

Paraffin embedded brain and spinal cord sections were assessed by immunohistochemistry for the presence of TMEV antigens, as described previously (31). Briefly, slides were deparaffinized with xylene and rehydrated in a graded ethanol series. The presence of virus antigen was determined by using a rabbit polyclonal antibody that reacts with TMEV capsid antigens (23). Either fluorescein isothiocyanate alone or biotinylated secondary antibodies were used to detect TMEV-specific antigen. The biotinylated anti-rabbit secondary was used with avidin biotin peroxidase complex (Vector Laboratories, Burlingame, CA, USA) and Hanker-Yates reagent to detect the virus antigen. Slides were counterstained with Gill’s hematoxylin.

### TMEV-specific enzyme linked immunosorbent assay

Purified TMEV was diluted in bicarbonate buffer and adsorbed to 96-well plates for 24 h before addition of serial serum dilutions from TMEV-infected transgenic mice. Biotinylated anti-mouse IgG and streptavidin labeled alkaline phosphatase (Jackson ImmunoResearch, West Grove, PA, USA) were used with p-nitrophenyl phosphate as a substrate to determine total TMEV-specific IgG. Absorbance was read at 405 nm. Antibodies to mouse IgG1, IgG2a, IgG2b, IgG3 and IgM (Sigma-Aldrich, St. Louis, MO, USA) were used to determine TMEV-specific isotypes. Anti-mouse HRP (Pierce Biotechnology, Rockford, IL, USA) was used with tetramethylbenzidine as the substrate for detection. Absorbances were read at 450 nm.

### Western blot for TMEV antigens

Overlapping proteins generated with the RTS 100 *E. coli* HY kit or whole virus were separated using SDS-PAGE. Samples were run on 12% polyacrylamide gels and blotted onto nitrocellulose membranes. Sera from infected mice were diluted (1:500) to determine specific reactivity of serum to TMEV antigens. We tested individual samples using the Surfblot apparatus (Idea Scientific, Minneapolis, MN, USA) which allows one to test individual serum samples without cutting the nitrocellulose membrane. Sera from recombinant VP2 immunized mice were diluted 1:50 to determine specific reactivity to TMEV. Secondary anti-mouse HRP (1:50 000) and the Pico Pro ECL substrate solution (Pierce Biotechnology) were used to detect reactive antibodies on autoradiographic film. Rabbit anti-TMEV and HRP labeled anti-Histag (BD Biosciences, San Jose, CA, USA) were used as positive controls and to verify the presence of whole virus and recombinant proteins on the blot. Sera from uninfected mice were used as negative controls.

### Immunization and lymphocyte proliferation assay

VP2 transgenic and non-transgenic mice were pre-bled and immunized with 200 µg of HisTag purified VP2 protein emulsified in complete Freund’s adjuvant. On day 10 mice were boosted with another 200 µg of VP2 protein. On day 21 of immunization mice were bled and sacrificed for proliferation assays. Spleens were harvested into RPMI media and were dissociated to release lymphocytes. Cells were washed and lysed with ACK buffer to remove red blood cells and washed. Cells were strained through mesh filters to remove non-cellular aggregates and then re-suspended in RPMI complete media containing polymyxin B to inhibit potential LPS contamination from protein extracts. Purified VP2 protein was added to culture in four serial dilutions. Cells were then incubated for 48 h before being pulsed with 1 µCi per well of ^3^H-thymidine and harvesting at 72 h. The cells were harvested onto glass fiber filter plates and allowed to dry before being read on a 96 well beta counter. Data were expressed as counts per minute.

## RESULTS

### The human ubiquitin c promoter drives expression of TMEV transgenes in FVB mice

**Figure 1:**
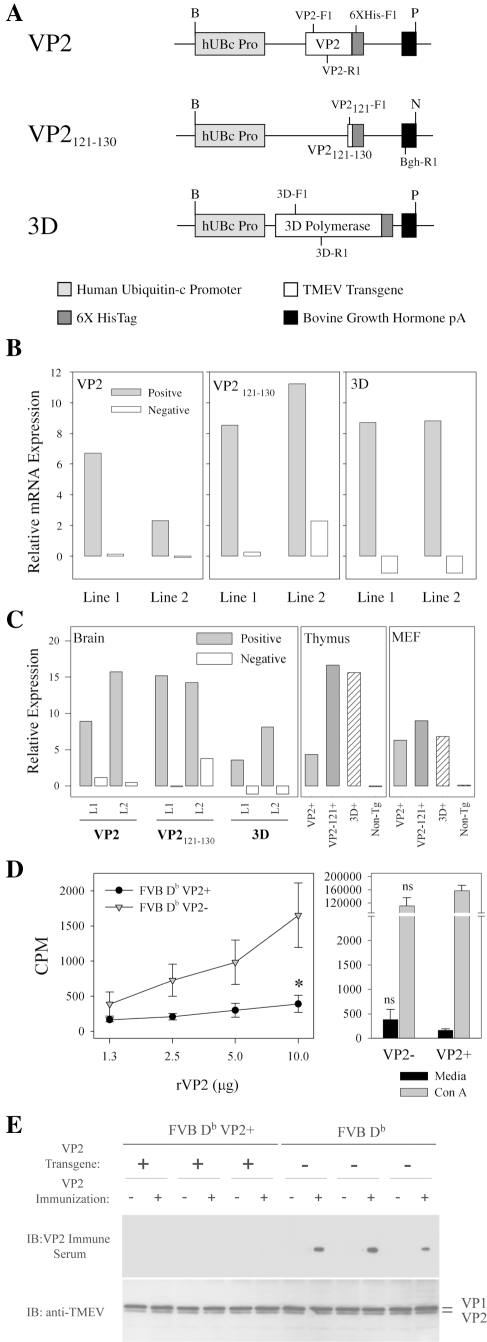
Generation of VP2, VP2_121-130_ and 3D transgenic mice and tolerance determination. Expression constructs were designed using the human ubiquitin c promoter **A.** Constructs containing cDNA for the capsid protein VP2, immunodominant CD8 antigen VP2_121-130_, and the 3D polymerase from TMEV were generated. 3′ flanking sequences for 6XHisTag and bovine growth hormone polyA were included. PCR primers for screening are marked. The following primer sequences were used to identify VP2 [VP2-F1(5′tggtcgactctg tggttacg) and VP2-R1(5′gccggtcttgcaaagatagt)], VP2_121-130_ [VP2_121_-F1(5′gccggctctcttcttgttt) and VP2_121_-R1(5′caagtggtgtccatggtgaa)] 3D [3D-F1(5′cgtagacatttccacaggatt) and 3D-R1(5′aa gacgttgtctttaccaa)] and any of the 3 [6XHis-F1(5′accggtcatcatcacc) and Bgh-R1(caccttc cagggtcaa)]. Restriction sites are identified as follows: B-BglII, P-PvuII and N-NspI. **B.** Relative transgene-specific expression in the brain of FVB VP2, FVB VP2_121-130_ and FVB 3D. Primers specific for the VP2, VP2_121-130_ and 3D transgenes were used to determine levels of mRNA transcript. **C.** Relative expression of transgenes in brain, thymus and mouse embryonic fibroblasts using primers specific for shared sequence motifs found in all transgenes. Primers used were 6XHis-F1 and Bgh-R1. Immune recognition of the self transgene was determined in FVB D^b^ VP2 transgenic mice using lymphocyte proliferation and western blot. **D.** Splenocytes from FVB D^b^ VP2+ mice failed to proliferate in response to VP2 antigen compared with FVB D^b^ VP2- mice (*significant by *t*-test *P* < 0.05). Sera from immunized mice were tested for reactivity to whole TMEV by western blot. **E.** No mice had immunoglobulin reactive to the TMEV-specific capsids VP1 or VP2 at pre-bleed (n = 6). FVB D^b^ VP2+ (n = 3) mice failed to generate VP2-specific immunoglobulin responses after immunization when compared with FVB D^b^ (n = 3) immune sera, which showed reactivity specifically to VP2. The western blot was stripped and re-probed with rabbit anti-TMEV to verify the presence of the TMEV proteins VP1 and VP2.

We generated expression constructs for the TMEV antigens VP2, VP2_121-130_ and 3D using the human ubiquitin c promoter (Figure 1A). This promoter has previously been used to induce broad tissue expression of other transgenes (33). We isolated total RNA from the brain and thymus of VP2, VP2_121-130_ and 3D transgenic mice to determine mRNA expression levels in these organs. Specific transgene expression in the brain was demonstrated in two lines of each by semi-quantitative RT-PCR (Figure 1B). To verify production of the full length RNA species, expression was further tested using primers that detected the shared 3′ fragment of the 3 expression constructs (Figure 1C). As thymic expression is paramount for tolerance induction we tested for expression of the transgenes in the thymus and demonstrated that all lines of mice expressed the transgene in this organ. We further tested fibroblasts grown from skin explants and verified expression in this cell type also (Figure 1C). Littermate control mice from all lines failed to demonstrate mRNA expression of the transgene in any organ or cell tested. Thus, we conclude that the VP2, VP2_121-130_ and 3D transgenes are expressed at the mRNA level in the brain and thymus. Further, brain homogenates from transgenic mice failed to show expression at the protein level as assessed by western blot. Our attempts to immunoprecipitate the antigen with an anti-HisTag antibody and probe for the antigen using TMEV anti-serum also failed to detect the proteins. In conclusion, despite the fact that mRNA was detected in multiple tissues, protein expression was not detected in any of the organs tested.

After establishing that transgene mRNAs were expressed in the FVB lines, we crossed these to FVB D^b^ mice, to generate a line that was on a TMEV-resistant background. All lines demonstrated transgene integration at levels similar to the FVB lines (data not shown). We used these FVB D^b^ transgenic strains for all subsequent experiments.

### The response to immunization with recombinant VP2 protein is lost in VP2 transgenic mice

As VP2 is expressed as a self antigen, we tested whether expression led to tolerance in FVB D^b^ VP2 mice. To test this we immunized FVB D^b^ VP2 transgenic mice and wild type mice with recombinant VP2 protein to determine whether reactivity to this viral antigen was altered. Splenocytes were isolated and re-challenged with VP2 protein *in vitro* to assess ^3^H-thymidine uptake after antigen pulse. VP2 transgenic mice, demonstrated a dramatic decrease in ^3^H-thymidine uptake when compared with non-transgenic controls (Figure 1D), demonstrating that there was a defect in the ability of lymphocytes to respond to this antigen. Further, both splenocyte populations proliferated in response to Con A, demonstrating that there was not an inherent defect in the splenocyte population. Therefore, we conclude that expression of the VP2 transgene as “self” led to a change in the ability of the T cells to respond to this antigen. In order to test whether antibody responses to recombinant VP2 were altered in FVB D^b^ VP2 mice, we tested pre-immune and immune serum from immunized mice for reactivity to TMEV. Not surprisingly, both FVB D^b^ and FVB D^b^ VP2 pre-immune sera failed to recognize the recombinant VP2 and the immune serum from FVB D^b^ mice reacted specifically to the VP2 capsid from the whole virus. This is in stark contrast to the FVB D^b^ VP2 mice which failed to generate an antibody response to recombinant VP2 (Figure 1E). Together these data demonstrate that there is a defect in the FVB D^b^ VP2 mice which prohibits the generation of an effective T-cell response to the VP2 protein and that this response parallels the loss of VP2-specific antibody after immunization with recombinant VP2.

### VP2 and VP2_121-130_ transgenic mice develop central nervous system demyelination after infection with TMEV

**Figure 2:**
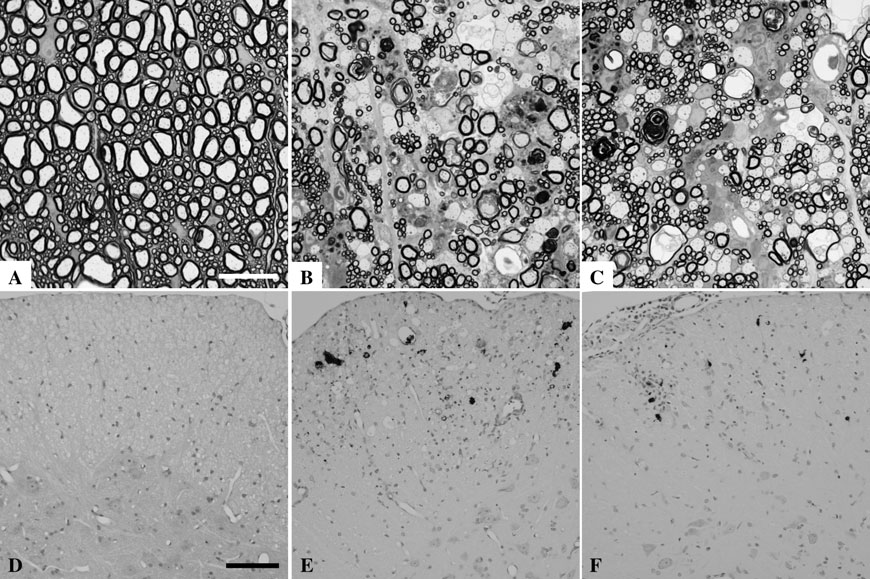
Demyelination and viral persistence in FVB-D^b^ VP2 and FVB-D^b^ VP2_121-130_ transgenic mice infected with TMEV for 45 days. Spinal cord sections from 45 day TMEV-infected mice were embedded in araldite, fixed with osmium tetroxide and stained with p-phenylenediamine to show demyelination in the white matter **A.** The resistant strain FVB D^b^ does not develop demyelination after TMEV infection. Note the tightly wrapped myelin around preserved spinal axons. **B.** Demyelination is abundant in the TMEV-infected FVB D^b^ VP2 transgenic mice, consistent with denuded axons and the infiltration of myelin filled macrophages. **C.** Similar spinal cord pathology is observed in the TMEV-infected FVB D^b^ VP2_121-130_ transgenic mice. **D.** TMEV antigen is absent from the spinal cord gray matter and white matter of FVB D^b^ mice at 45 days post TMEV infection. **E.** Viral antigen is present in the spinal cord of FVB D^b^ VP2 transgenic mice. Virus staining is consistent with infection of glial cells or macrophages in the white matter and lack of virus antigen in the gray matter of the spinal cord. **F.** Virus staining in FVB D^b^ VP2_121-130_ mice infected with TMEV for 45 days is similar to that observed in FVB D^b^ VP2 mice. Bar in (**A**) represents 10 µm for (**A–C**). Black bar in (**D**) represents 50 µm for (**D–F**).

Having demonstrated that VP2, VP2_121-130_, and 3D transgene-specific mRNA was expressed in the brain and thymus and that this expression level could lead to tolerance we next tested whether expression of these transgenes in H-2D^b^ mice could prevent the clearance of TMEV from the CNS. We infected transgenic mice with TMEV for 45 days and analyzed spinal cord sections for the presence of pathology. We analyzed the susceptible strain FVB (H-2^q^), resistant strain FVB D^b^ (H-2^b^) and the TMEV transgenic strains FVB D^b^ VP2, FVB D^b^ VP2_121-130_ and FVB-D^b^ 3D. After 45 days of infection with TMEV, FVB mice developed pathology that was characterized by focal areas of demyelination with axons that had completely or partially lost their myelin sheaths along with infiltration of immune cells into the spinal cord parenchyma (Table 1). In contrast, this phenotype was lost when the H-2D^b^ transgene was expressed on the FVB background (Table 1, Figure 2A). These mice failed to develop the pathology observed in the FVB strain. This is consistent with previous data demonstrating that susceptibility to TMEV-induced demyelination is restricted by H-2D^b^(29). Expression of the 3D transgene resulted in minimal or no demyelination in these strains (Table 1). However, expression of the complete VP2 capsid protein resulted in a loss of resistance to demyelination (Table 1, Figure 2B). Similarly, transgenic mice that expressed the immunodominant CD8+ T-cell antigen VP2_121-130_ also lost resistance to TMEV-induced demyelination (Figure 2C). Further, the quality and extent of demyelination seen in both strains was not different (Table 1, Figure 2B,C). This finding clearly demonstrates that expression of the VP2 and VP2_121-130_ transgenes leads to the development of demyelination in the spinal cord following TMEV infection.

**Table 1:** Spinal cord pathology in TMEV-infected transgenic mice.

				Percent of spinal cord quadrants with disease (mean ± SEM)		
				
Transgenic	H-2	Number of mice	Frequency of demyelination	Gray matter inflammation	Meningeal inflammation	Demyelination
FVB	*q*	10	70%	1.0 ± 0.5	12.3 ± 3.1	12.4 ± 4.3
FVB-D^b^	*b*	10	10%	0.0 ± 0.0	1.5 ± 1.0	0.6 ± 0.6
FVB-D^b^ 3D	*b*	8	0%	0.0 ± 0.0	0.0 ± 0.0	0.0 ± 0.0
FVB-D^b^ VP2	*b*	5	100%	1.6 ± 0.8	21.3 ± 4.0	20.4 ± 4.1
FVB-D^b^ VP2_121-130_	*b*	6	100%	0.4 ± 0.4	26.1 ± 4.7	18.4 ± 4.2

### Demyelination is paralleled by the presence of viral antigen in VP2 and VP2_121-130_ transgenic mice

TMEV-infection induced demyelination is most often accompanied by the presence of viral antigen in the spinal cord. Thus, we performed immunohistochemistry on spinal cord sections to test for the presence of TMEV antigen in 45 day infected mice. We analyzed 10 sections from each of the infected mice. None of the FVB D^b^ (n = 10) mice had TMEV antigen positive cells in any of the spinal cord sections. These mice consistently cleared virus as demonstrated by the lack of virus antigen staining in all of the spinal cord sections analyzed (Figure 2D). In contrast all FVB D^b^ VP2 (*n* = 5; *P* < 0.001 by Fisher Exact Test) and all FVB D^b^ VP2_121-130_ (*n* = 6; *P* < 0.001 by Fisher exact test) mice developed persistent antigen after 45 days of TMEV infection. These strains consistently demonstrated virus antigen in the spinal cord white matter, often associated with inflammatory cells and parenchymal damage (Figure 2E,F). We conclude from this that the presence of the VP2 or VP2_121-130_ transgenes in TMEV-infected mice resulted in viral persistence that was associated with demyelination.

### Expression of VP2 and VP2_121-130_ transgenes does not alter the antibody responses to TMEV

**Figure 3:**
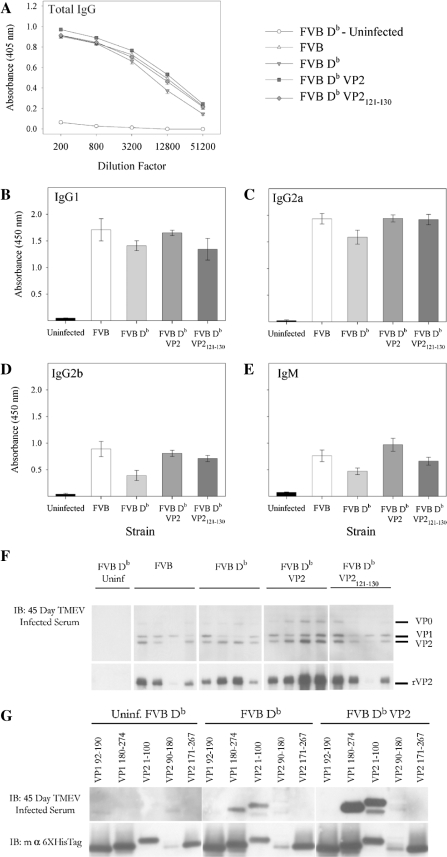
Total IgG- and isotype-specific antibody responses to TMEV from FVB-D^b^ VP2 and FVB-D^b^ VP2_121-130_ transgenic mice infected with TMEV for 45 days. Serum from 45 day infected mice was collected and assayed for recognition of TMEV and its specific antibody epitopes by ELISA and western blot **A.** Total IgG specific for plate bound TMEV was not different between any of the 45-day infected strains tested. The specific isotype reactivity to TMEV were also tested. The predominant isotype responses to TMEV in infected mice were (**B**) IgG1 and (**C**) IgG2a. (**D**) IgG2b and (**E**) IgM responses were also present at 45 days post TMEV infection. No differences were observed between strains. **F.** TMEV blot for reactivity of 45 day infected serum to specific capsid proteins. Three of four FVB mice demonstrated specific reactivity to VP2, whereas four of four FVB D^b^ mice generated antibody that recognized VP2. FVB D^b^ VP2 and FVB D^b^ VP2_121-130_ mice also generated VP2-specific antibody (4/4 and 3/4). **G.** FVB D^b^ and FVB D^b^ VP2 mice recognize similar VP1 and VP2 antibody epitopes.

Having established that resistance to TMEV-induced demyelination and viral persistence was abrogated, we next asked if specific immune responses to TMEV were altered and contributed to this phenotype. We first looked at TMEV-specific B cell responses, particularly the generation of TMEV-specific antibody and the isotypes generated after infection. Sera from infected mice were tested by ELISA. Virus-specific antibody titers did not differ between the strains of mice that had demyelination. FVB, FVB-D^b^ VP2 and FVB-D^b^ VP2_121-130_ all had similar TMEV-specific IgG responses. However, the resistant strain, FVB-D^b^, had a slightly reduced titer (Figure 3A), consistent with viral clearance and lack of persistent antigen.

Further, to determine more specifically the differences in isotypes between transgenic and non-transgenic mice in response to TMEV infection we analyzed antibody responses to TMEV. We analyzed IgG1, IgG2a, IgG2b, IgG3 and IgM specific TMEV responses from TMEV-infected mice. No differences were observed in these specific responses between the FVB, FVB-D^b^ VP2 and FVB-D^b^ VP2_121-130_ mice (Figure 3B–E). Here again the TMEV-infected FVB-D^b^ mice had a slightly reduced antibody response, as observed for all isotypes.

We then tested the antigen specificity of the IgG response generated during TMEV infection in serum from TMEV-infected FVB, FVB D^b^, FVB D^b^ VP2 and FVB D^b^ VP2_121-130_ mice by western blot. All strains demonstrated reactivity to VP1 and VP2 (Figure 3F), demonstrating that the specificity of the antibody response was not different. To further verify reactivity to specific epitopes we tested binding of antibody to overlapping proteins from VP1 and VP2. Data from this experiment revealed that TMEV-infected FVB D^b^ VP2 and FVB D^b^ strains both generated antibody responses to epitopes in VP1 (180–274) and VP2 (1–100) (Figure 3G). This is consistent with previous findings which showed that antibody responses to these antigens are found during TMEV infection (11). Taken together these data demonstrate that the ability of B cells to recognize VP2 antigen during TMEV infection is not altered when VP2 is expressed as a self antigen.

### Decrease in brain infiltrating CD8*+* T cells in VP2 and VP2_121-130_ transgenic mice

**Figure 4:**
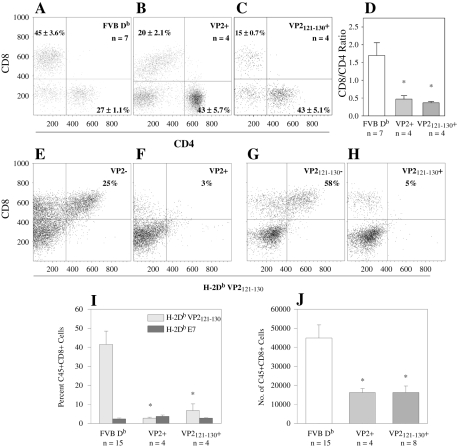
Loss of VP2_121-130_ specific CD8+ T cells from brain infiltrates of FVB-D^b^ VP2 and FVB-D^b^ VP2_121-130_ transgenic mice infected with TMEV for 7 days. Brain infiltrating lymphocytes were isolated from mice and assessed for the presence of CD4, CD8 and H-2D^b^/VP2_121-130_ tetramer positive cells **A.** FVB D^b^ mice demonstrate a preponderance of CD8+ cells among the CD45+ population with 45 + 3.6% of the CD45+ population being CD8+. **B.** FVB D^b^ VP2 transgenic mice showed a decrease in the percent of CD8+ cells infiltrating the brain (20 + 2.1%). **C.** FVB D^b^ VP2_121-130_ transgenic mice showed a similar decrease in the percent of brain infiltrating CD8+ cells (15 ± 0.7%). **D.** The ratio of percent CD8+ to percent CD4+ was significantly reduced in FVB D^b^ VP2 and FVB D^b^ VP2_121-130_ transgenic mice when compared with FVB D^b^ control (*significant by *t*-test *P* < 0.001). Representative flow cytometry samples of brain infiltrating lymphocytes from FVB D^b^ VP2+ and FVB D^b^ VP2- littermate control. **E.** 25% of the CD8+ population was positive for H-2D^b^/VP2_121-130_ tetramer in the FVB D^b^ VP2- control isolate. **F.** Very few H-2D^b^/VP2_121-130_ tetramer positive cells were found in the FVB D^b^ VP2+ isolate. Similar results were found in the FVB D^b^ VP2_121-130_ transgenic mice. **G.** VP2_121-130_ negative mice showed a majority of CD8+ cells being positive for the VP2_121-130_ tetramer. **H.** FVB D^b^ VP2_121-130_ mice had a dramatic decrease in tetramer positive CD8 cells. **I.** Decreased H-2D^b^/VP2_121-130_ positive CD8 cells in FVB D^b^ VP2 and FVB D^b^ VP2_121-130_ transgenic brain infiltrating lymphocytes when compared with control (*Significant by Mann–Whitney Rank Sum Test *P* < 0.05). No significant differences were found between H-2D^b^/VP2_121-130_ positive CD8 cells and H-2D^b^/E7 positive CD8 cells in VP2 and VP2_121-130_ transgenic mice (Mann–Whitney Rank Sum Test, *P* = 0.229 and *P* = 0.886). Absolute numbers of CD45Hi and CD8+ cells in the brain isolates were calculated from the total cells isolated and flow cytometry data. **J.** No significant differences were observed in CD45Hi numbers between FVB D^b^ and FVB D^b^ VP2 (*P* = 0.546) or between FVB D^b^ and FVB D^b^ VP2_121-130_ (*P* = 0.455). Both the FVB D^b^ VP2 and FVB D^b^ VP2_121-130_ transgenic mice had significantly reduced numbers of CD8 cells infiltrating the brain (*significant by *t*-test *P* = 0.011 and *P* = 0.001).

We next chose to determine the effect of VP2 and VP2_121-130_ transgene expression on CD4 and CD8 T-cell populations that infiltrate the brain of TMEV-infected animals. We isolated brain and spinal cord infiltrating lymphocytes (BILs) from 7-day infected VP2 and VP2_121-130_ transgenic mice, as well as from non-transgenic mice and analyzed them by flow cytometry. Lymphocyte populations were first identified by gating on the CD45+ common leukocyte antigen to distinguish lymphocytes from non-hematopoietic cells. These cells were subsequently analyzed for CD4 and CD8 populations. On average, CD4+ and CD8+ cells accounted for 66 ± 3% of the CD45+ population infiltrating the brain. However, when these populations are analyzed separately, differences between transgenic and non-transgenic mice became obvious. In non-transgenic mice the CD8+ population accounted for 45 ± 3.6% of the total CD45+ cells that infiltrate the brain, whereas the CD4+ population was 27 ± 1.1% of this population (Figure 4A). In contrast, the VP2 and VP2_121-130_ transgenic mice showed decreases in the percent of CD8+ cells infiltrating the brain (20 ± 2.1% and 15 ± 0.7% respectively) and increases in the percent of CD4+ cells (43 ± 5.7% and 43 ± 5.1% respectively) after 7 days of infection with TMEV (Figure 4B,C). Thus, the representation of CD8+ and CD4+ cells was reversed and the ratio of CD8+ to CD4+ was significantly reduced in mice expressing VP2 or VP2_121-130_ as compared with non-transgenic mice (Figure 4D), indicating that the presence of these transgenes altered the magnitude of the CD8+ T-cell response in the brain. We then analyzed the number of CD8+ T cells infiltrating the brain of TMEV-infected mice. Although the total number of cells harvested from the brains of transgenic mice had a tendency to be lower than non-transgenic mice, this did not reach statistical significance. This may be caused by the complexity of the cell infiltrate isolated from brain homogenates which may include brain resident cells. Therefore, we gated on the CD45Hi population to determine more precisely the numbers of lymphocyte subsets in the brain infiltrate. We recovered 106 312 ± 16 532 CD45Hi cells from the FVB D^b^ infected strain and 85 924 ± 12 902 and 86 658 ± 17 111 from the FVB D^b^ VP2 and FVB D^b^ VP2_121-130_ respectively. There were no statistical differences between the groups (*P* = 0.546 and *P* = 0.455 by *t*-test) (Figure 4J). However, when the number of CD8+ cells among the CD45+ high population was calculated, statistically significant differences were seen. We found 44 884 ± 6952 CD8+ cells in the brains of the control FVB D^b^ mice (Figure 4J). In contrast, the FVB D^b^ VP2 and FVB D^b^ VP2_121-130_ mice had CD8 numbers that were significantly lower (Figure 4J), 16 218 ± 2090 (*P* = 0.011, Mann–Whitney Rank Sum) and 16 298 ± 3435 (*P* = 0.001, Mann–Whitney Rank Sum). Therefore, we conclude that there are decreased numbers of CD8+ cells infiltrating the brains of 7-day infected FVB D^b^ VP2 and FVB D^b^ VP2_121-130_ transgenic mice.

### VP2_121-130_ specific CD8*+* T cells are absent in brain infiltrates of 7 day infected VP2 and VP2_121-130_ transgenic mice

It has been previously shown that viral clearance in H-2D^b^ mice is dependent on mounting a CTL response to the H-2D^b^ bound VP2_121-130_ peptide from TMEV (20). Having established that there was a decrease in the number of CD8+ T cells infiltrating the CNS, we asked whether this is caused by the specific loss of CD8+ T cells that recognize the immunodominant VP2_121-130_ epitope. We used H-2D^b^/VP2_121-130_ tetramers to determine the presence of antigen-specific cells in CNS infiltrates. H-2D^b^ peptide E7 from human papillomavirus served as a control for non-specific binding. We found that 3.1 ± 0.4% of all CD8+ cells from all groups were positive by staining for E7-specific tetramer, and no statistically significant differences were seen between the groups (data not shown). In contrast, the H-2D^b^/VP2_121-130_ specific populations made up 42 ± 7% of the CD8+ T-cell populations in infected FVB D^b^ control mice (Figure 4I). Representative samples are shown for VP2 negative and VP2_121-130_ negative (Figure 4E,G) littermate controls. Importantly, FVB D^b^ VP2 and FVB D^b^ VP2_121-130_ transgenic mice demonstrated a profound reduction in the percent of VP2_121-130_ specific CD8+ T cells (Figure 4F,H). Moreover, this reduction in antigen-specific CD8+ cells was equivalent to the numbers of E7 antigen-specific CD8+ cells (Figure 4I). We conclude that the H-D^b^/ VP2_121-130_ specific response was lost in both FVB D^b^ VP2 and FVB D^b^ VP2_121-130_ transgenic mice after infection with TMEV.

### The severity of brain pathology is not altered during acute infection in VP2_121-130_ transgenic mice

**Figure 5:**
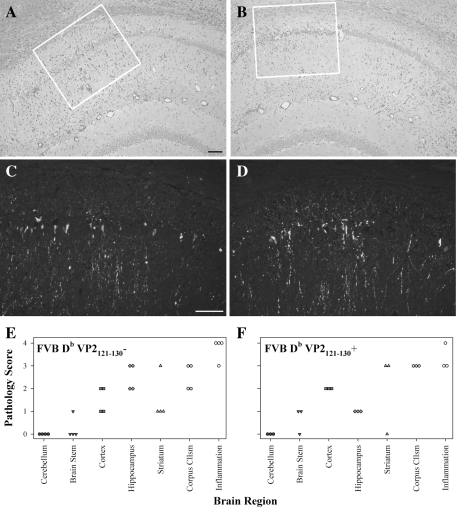
Brain pathology and acute viral infection in FVB-D^b^ VP2_121-130_ transgenic mice infected with TMEV for 7 days. Paraffin-embedded brain sections were stained with hemotoxylin and eosin or TMEV-specific antibody to assess severity of brain pathology and presence of viral antigen at the site of pathology **A.** Transgene negative littermate control mice demonstrate a severe hippocampal pathology that is accompanied by a robust inflammatory infiltrate (10×). **B.** FVB-D^b^ VP2_121-130_ transgenic mice acquire a similar pathology; however, the inflammatory infiltrate is not as extensive in the hippocampal region (10×). **C.** Viral antigen staining in an area corresponding to the white box in A. Virus positive cells are consistently found in this area as demonstrated by the staining of neuronal cell bodies and processes (20×). **D.** Virus antigen was assessed in an area corresponding to the white box in B and which is anatomically similar to that observed in C. Similar viral staining patterns are observed in FVB-D^b^ VP2_121-130_ transgenic mice, when compared with the littermate control (20×). Pathology was scored on a 4 point scale to assess the extent of brain pathology. **E.** FVB-D^b^ VP2_121-130_ negative littermate controls had moderate to severe pathology in the cortex, hippocampus, striatum and corpus collosum, this was accompanied by extensive meningeal inflammation. **F.** FVB-D^b^ VP2_121-130_ mice demonstrate similar pathology with decreased inflammation in the hippocampus. Bar in (**A**) represents 50 µm for (**A,B**) and bar in (**C**) represents 50 µm for (**C,D**).

To determine whether the loss of virus-specific CD8+ T cells had an effect on the severity of pathology observed in the brain after 7 days of infection, we assessed the pathology in brains isolated from FVB D^b^ VP2_121-130_ mice and in transgene negative littermate controls (Figure 5A,B). Pathology in both strains consisted of moderate to severe parenchymal damage in the cortex, hippocampus, striatum and corpus collosum. These areas also consistently showed inflammatory infiltrates with perivascular cuffs most noticeably present in the hippocampus. Furthermore, hippocampal neurons consistently demonstrated neuronophagia and vacuolation, consistent with damage to these cells. To determine whether these cells were infected with TMEV we stained serial sections from those represented in Figures 5A and B for virus antigen. We found viral antigen in many neuronal cell bodies and processes found in this area (Figure 5C,D) demonstrating the association of virus with brain pathology. The primary site of infection, however, was the hippocampus; this was observed in the FVB D^b^ VP2_121-130_, as well as the littermate controls. Finally, we used a 4-point scoring system to quantitatively assess the pathology observed in the brains of infected mice. No differences were observed in any of the regions assessed (Figure 5E,F). Although there were no statistical differences observed in any of these regions, the scores in the hippocampal region in the FVB D^b^ VP2_121-130_ were consistently lower than the littermate control and did approach significance (*P* = 0.057, Mann–Whitney Rank Sum). Consequently, the slight difference in pathology score is most likely because of the presence of an increased inflammatory infiltrate rather than parenchymal damage, as there is a decrease in the number of CD8+ T cells that infiltrate the brain of FVB D^b^ VP2_121-130_ mice (Figure 4J). We conclude that expression of the VP2_121-130_ transgene does not influence the extent of brain pathology observed in 7-day TMEV-infected mice.

## DISCUSSION

In this study we have shown that the transgenic expression of a complete virus capsid protein, VP2 or the specific CD8 T-cell epitope VP2_121-130_ leads to deletion of this response from the T-cell repertoire. This loss was apparent during a viral infection that typically leads to a robust CD8 T cell infiltrate into the brains of mice infected with TMEV. Additionally, immunization with recombinant VP2 was also defective in mice expressing the complete VP2 capsid protein, suggesting that other epitopes from VP2 may also be deleted. Further, the data support the conclusion that the VP2_121-130_ specific CD8 response is deleted from the T-cell repertoire in both of these transgenic lines.

One shortfall to this study was that we were unable to demonstrate the expression of VP2 or VP2_121-130_ protein in these transgenic lines. This finding, however, was not surprising considering that expression levels of protein necessary for the induction of tolerance may not be detected by the methods we chose. In support of this possibility, others have shown that tolerance can be induced by levels of protein that are not detectable by these methods (40) and that very low levels of protein expression can be detected by MHC-restricted lymphocytes (25). Further, because these proteins are being expressed in an environment that is not conducive to normal VP2 folding, such as during viral infection, these proteins may be directly targeted for degradation. This would be analogous to the defective ribosomal products (DRiPs) described by Yewdell et al (45). However, these proteins are expressed at a sufficient level to promote deletion of a VP2 or VP2_121-130_ specific T cells.

As these transgenes were expressed under the ubiquitin promoter, one could surmise that transgene expression itself caused that pathology demonstrated and that viral infection triggered an autoimmune response directed against cells in the CNS. Although appealing, we feel that this is not a possibility because the pathology is consistent with that seen in other susceptible strains of mice such as FVB. In addition, no outward signs of systemic autoimmunity were evident in any of the animals tested. Additionally, acute infection of the brain demonstrated a similar pattern of pathology and viral infection, with a concomitant decrease in infiltrating lymphocytes, further supporting the conclusion that there is not an autoimmune component to this model during acute infection. Further, initial infection with TMEV is localized to the gray matter of the brain and spinal cord with subsequent clearance in mice of susceptible and resistant haplotypes. Importantly, none of the VP2 or VP2_121-130_ transgenic mice showed viral persistence in the gray matter of the spinal cord (Figure 2D–F). In the event that T cells were recruited to the CNS that recognized the VP2 transgenic antigen, one would expect to see a non-discriminant pattern of pathology because of the universal expression of the transgene rather than focal demyelinating lesions. Therefore, we conclude that the data favor the hypothesis that transgene expression leads to the development of spinal cord pathology and viral persistence because of the loss of an antigen-specific immune effector function.

Another interesting finding in this study was that VP2-specific antibody responses were not detected in response to immunization with recombinant VP2, but were detected in response to viral infection. Although initially this finding seems contradictory, we feel that it further supports the conclusion that the antigen-specific deletion is distinctively the loss of a T-cell response rather than a B cell response. One possibility is that during viral infection B cells specific for VP2 phagocytose the complete TMEV virion and present another non-VP2-specific class II epitope to CD4+ helper cells, thereby providing the necessary CD4 helper response necessary for maturation of the antibody response. As no other epitopes are available other than VP2 during the immunization with recombinant protein, no CD4 help is available because they are tolerized to the complete VP2 antigen. This possibility could be further tested by crossing VP2 transgenic mice to mice expressing other TMEV determinants, such as VP1 and infecting with TMEV. Another possibility for the generation of an antibody response during infection is that several examples of T cell-independent antiviral antibody responses have been identified (36), such as that observed during TMEV infection. This is further supported by data using the TMEV model that demonstrated both CD4 and CD8 knockout mice infected with TMEV for 45 days generated TMEV-specific IgG at similar levels (22). Finally, this observation could be explained by the loss of tolerance to the VP2 antigen in response to TMEV infection. In contrast to current data demonstrating that tolerance can be overcome by viral infection (21), we favor the hypothesis that T cell-specific tolerance was maintained. We have demonstrated that VP2_121-130_ specific CD8 T cells were absent from the brain infiltrates of TMEV-infected mice by staining with MHC I tetramers. As these transgenes were expressed in the thymus, deletion occurred during thymic development and hence VP2 reactive cells were deleted from the repertoire. Because the mechanism of positive selection occurs at the double positive stage (35), we favor the possibility that all T cells are selected on the VP2 antigen and are deleted from the active repertoire independent of CD4 or CD8 expression. Granted, some positively selected T cells may develop into CD4 regulatory cells. However, data suggest that few T cells escape negative selection (18). Therefore, the data support the hypothesis that the VP2-specific T-cell responses are deleted from the repertoire and that the B cell response is only affected in response to VP2 when it depends on T cell help through VP2.

Previous studies have identified epitopes within the VP2 region of TMEV that are important for the resistance to and development of pathology (9, 20). These include the CD8 epitope VP2_121-130_ which is critical for viral clearance in resistant mice and the CD4 epitope VP2_74-86_ which has been shown to contribute to TMEV-induced demyelination in susceptible strains of mice. Our current model is unique in that both of these epitopes are represented in the FVB D^b^ VP2 strain and only one is represented in the FVB D^b^ VP2_121-130_ strain. This allows us to compare the contribution of the immune response outside of the VP2_121-130_ epitope. Our pathology data demonstrate that there were no significant differences in the demyelination or inflammatory infiltrates observed in the two strains after 45 days of TMEV infection, leading to the conclusion that epitopes outside of the VP2_121-130_ epitope do not contribute to the enhancement of pathology observed in this model. As this strain was developed on the FVB (H-2^q^) background it is also possible that other VP2 epitopes are not recognized in this strain and that the VP2_74-86_ epitope is specifically recognized by the SJL mouse. Our data suggests that immunization with VP2 can lead to a memory response to the whole VP2 antigen. This finding suggests that there may potentially be CD4-specific VP2 responses deleted in addition to the CD8 response to VP2_121-130_. However, we do not believe that these VP2-specific responses make a significant contribution to pathology because previous studies in susceptible SJL mice have demonstrated that T cells reactive to VP1 secrete fourfold to fivefold more IFN-γ than VP2 reactive cells in response to *in vitro* stimulation (44). Future studies that definitively determine whether additional CD8 and CD4 T-cell epitopes are recognized will be important in determining their contribution to TMEV-induced pathology in this model.

A final point of interest in this work involves the brain pathology observed in the 7-day acute infection seen in the FVB D^b^ VP2_121-130_ strain. The VP2_121-130_ transgenic mice had a significant decrease in the number of CD8 T cells in the brain and this appeared to correlate with a decrease in hippocampal pathology, although not significant by statistical measures. In contrast, the littermate control had a robust CD8 infiltrate which corresponded to a population of cells that specifically recognized the VP2_121-130_ peptide on H-2D^b^. Knowing that the FVB D^b^ VP2_121-130_ transgenic ultimately develops viral persistence, it is not surprising that the apparent sparing of brain tissue is at the expense of chronic demyelinating disease and viral persistence in the spinal cord, whereas the littermate control can efficiently eliminate virus because of the presence of virus-specific T cells. Of interest will be to further understand whether the CD8 cells specific for the VP2_121-130_ peptide are contributing to death or injury in cells that are or are not infected with the virus or whether the lack of VP2_121-130_ specific CD8 cells leads to an increase in cell death caused by viral infection. Answers to these questions will further our understanding of the consequences of epitope-specific responses observed neurologic disease.

Our data support the hypothesis that the expression of VP2 and VP2_121-130_ transgenes specifically deletes T cells reactive to those expressed antigens and that this effect is likely T cell specific, as B-cell responses to VP2 can be detected. Further, we show that expression of a construct that encodes only 20 amino acids of the VP2 gene was able to specifically delete an immunodominant CD8 T-cell response during a virus infection of the CNS. In addition, expression of the full VP2 protein yielded a similar result demonstrating that epitopes outside of the VP2_121-130_ epitope do not contribute to pathology. This model will have important implications for the understanding of T-cell deletion and tolerance as well as having implications for therapeutic interventions involving tolerance induction.

These experiments do not address the mechanisms of how demyelination is occurring in FVB D^b^ VP2 or FVB D^b^ VP2_121-130_ mice. However, they do demonstrate that deletion of the immune response to VP2 is sufficient to convert a mouse of a resistant haplotype to a mouse that is susceptible to demyelination. Demyelination may be the result of (i) direct virus infection of oligodendrocytes independent of the host T-cell response (32), (ii) CD4 or CD8 T cells directed to antigens not tolerized by these transgenes (37, 42, 43), or (iii) epitope spreading to myelin antigens (21). Further experiments will be necessary to distinguish between these possibilities.

## References

[1] Baldwin TA, Hogquist KA, Jameson SC (2004). The fourth way? Harnessing aggressive tendencies in the thymus. J Immunol.

[2] Bielekova B, Goodwin B, Richert N, Cortese I, Kondo T, Afshar G, Gran B, Eaton J, Antel J, Frank JA, McFarland HF, Martin R (2000). Encephalitogenic potential of the myelin basic protein peptide (amino acids 83–99) in multiple sclerosis: results of a phase II clinical trial with an altered peptide ligand. Nat Med.

[3] Borson ND, Paul C, Lin X, Nevala WK, Strausbauch MA, Rodriguez M, Wettstein PJ (1997). Brain-infiltrating cytolytic T lymphocytes specific for Theiler’s virus recognize H2Db molecules complexed with a viral VP2 peptide lacking a consensus anchor residue. J Virol.

[4] Clatch RJ, Pevear DC, Rozhon E, Roos RP, Miller SD, Lipton HL (1987). Characterization and specificity of humoral immune responses to Theiler’s murine encephalomyelitis virus capsid proteins. J Gen Virol.

[5] Critchfield JM, Racke MK, Zuniga-Pflucker JC, Cannella B, Raine CS, Goverman J, Lenardo MJ (1994). T cell deletion in high antigen dose therapy of autoimmune encephalomyelitis. Science.

[6] Disis ML, Salazar LG, Knutson KL (2004). Peptide-based vaccines in breast cancer. Breast Dis.

[7] Drescher KM, Murray PD, David CS, Pease LR, Rodriguez M (1999). CNS cell populations are protected from virus-induced pathology by distinct arms of the immune system. Brain Pathol.

[8] Ferguson TA, Stuart PM, Herndon JM, Griffith TS (2003). Apoptosis, tolerance, and regulatory T cells–old wine, new wineskins. Immunol Rev.

[9] Gerety SJ, Rundell MK, Dal Canto MC, Miller SD (1994). Class II-restricted T cell responses in Theiler’s murine encephalomyelitis virus-induced demyelinating disease. VI. Potentiation of demyelination with and characterization of an immunopathologic CD4+ T cell line specific for an immunodominant VP2 epitope. J Immunol.

[10] Hogquist KA, Baldwin TA, Jameson SC (2005). Central tolerance: learning self-control in the thymus. Nat Rev Immunol.

[11] Inoue A, Choe YK, Kim BS (1994). Analysis of antibody responses to predominant linear epitopes of Theiler’s murine encephalomyelitis virus. J Virol.

[12] Johnson AJ, Njenga MK, Hansen MJ, Kuhns ST, Chen L, Rodriguez M, Pease LR (1999). Prevalent class I-restricted T-cell response to the Theiler’s virus epitope Db:vP2121-130 in the absence of endogenous CD4 help, tumor necrosis factor alpha, gamma interferon, perforin, or costimulation through CD28. J Virol.

[13] Johnson AJ, Mendez-Fernandez Y, Moyer AM, Sloma CR, Pirko I, Block MS, Rodriguez M, Pease LR (2005). Antigen-specific CD8+ T cells mediate a peptide-induced fatal syndrome. J Immunol.

[14] Kang B, Kang HK, Kim BS (2005). Identification of capsid epitopes of Theiler’s virus recognized by CNS-infiltrating CD4+ T cells from virus-infected C57BL/6 mice. Virus Res.

[15] Kappos L, Comi G, Panitch H, Oger J, Antel J, Conlon P, Steinman L (2000). Induction of a non-encephalitogenic type 2 T helper-cell autoimmune response in multiple sclerosis after administration of an altered peptide ligand in a placebo-controlled, randomized phase II trial. The Altered Peptide Ligand in Relapsing MS Study Group. Nat Med.

[16] Lin X, Njenga MK, Johnson AJ, Pavelko KD, David CS, Pease LR, Rodriguez M (2002). Transgenic expression of Theiler’s murine encephalomyelitis virus genes in H-2(b) mice inhibits resistance to virus-induced demyelination. J Virol.

[17] Lindsley MD, Thiemann R, Rodriguez M (1991). Cytotoxic T cells isolated from the central nervous systems of mice infected with Theiler’s virus. J Virol.

[18] Lohr J, Knoechel B, Nagabhushanam V, Abbas AK (2005). T-cell tolerance and autoimmunity to systemic and tissue-restricted self-antigens. Immunol Rev.

[19] McDevitt H (2004). Specific antigen vaccination to treat autoimmune disease. Proc Natl Acad Sci USA.

[20] Mendez-Fernandez YV, Johnson AJ, Rodriguez M, Pease LR (2003). Clearance of Theiler’s virus infection depends on the ability to generate a CD8+ T cell response against a single immunodominant viral peptide. Eur J Immunol.

[21] Miller SD, Vanderlugt CL, Begolka WS, Pao W, Yauch RL, Neville KL, Katz-Levy Y, Carrizosa A, Kim BS (1997). Persistent infection with Theiler’s virus leads to CNS autoimmunity via epitope spreading. Nat Med.

[22] Murray PD, Pavelko KD, Leibowitz J, Lin X, Rodriguez M (1998). CD4(+) and CD8(+) T cells make discrete contributions to demyelination and neurologic disease in a viral model of multiple sclerosis. J Virol.

[23] Njenga MK, Pavelko KD, Baisch J, Lin X, David C, Leibowitz J, Rodriguez M (1996). Theiler’s virus persistence and demyelination in major histocompatibility complex class II-deficient mice. J Virol.

[24] Ohara Y, Stein S, Fu JL, Stillman L, Klaman L, Roos RP (1988). Molecular cloning and sequence determination of DA strain of Theiler’s murine encephalomyelitis viruses. Virology.

[25] Oldstone MB, Nerenberg M, Southern P, Price J, Lewicki H (1991). Virus infection triggers insulin-dependent diabetes mellitus in a transgenic model: role of anti-self (virus) immune response. Cell.

[26] Pavelko KD, Howe CL, Drescher KM, Gamez JD, Johnson AJ, Wei T, Ransohoff RM, Rodriguez M (2003). Interleukin-6 protects anterior horn neurons from lethal virus-induced injury. J Neurosci.

[27] Pedotti R, Sanna M, Tsai M, DeVoss J, Steinman L, McDevitt H, Galli SJ (2003). Severe anaphylactic reactions to glutamic acid decarboxylase (GAD) self peptides in NOD mice that spontaneously develop autoimmune type 1 diabetes mellitus. BMC Immunol.

[28] Prakken BJ, Samodal R, Le TD, Giannoni F, Yung GP, Scavulli J, Amox D, Roord S, de Kleer I, Bonnin D, Lanza P, Berry C, Massa M, Billetta R, Albani S (2004). Epitope-specific immunotherapy induces immune deviation of proinflammatory T cells in rheumatoid arthritis. Proc Natl Acad Sci USA.

[29] Rodriguez M, Leibowitz J, David CS (1986). Susceptibility to Theiler’s virus-induced demyelination. Mapping of the gene within the H-2D region. J Exp Med.

[30] Rodriguez M, Oleszak E, Leibowitz J (1987). Theiler’s murine encephalomyelitis: a model of demyelination and persistence of virus. Crit Rev Immunol.

[31] Rodriguez M, Zoecklein LJ, Howe CL, Pavelko KD, Gamez JD, Nakane S, Papke LM (2003). Gamma interferon is critical for neuronal viral clearance and protection in a susceptible mouse strain following early intracranial Theiler’s murine encephalomyelitis virus infection. J Virol.

[32] Rosenthal A, Fujinami RS, Lampert PW (1986). Mechanism of Theiler’s virus-induced demyelination in nude mice. Lab Invest.

[33] Schorpp M, Jager R, Schellander K, Schenkel J, Wagner EF, Weiher H, Angel P (1996). The human ubiquitin C promoter directs high ubiquitous expression of transgenes in mice. Nucleic Acids Res.

[34] Sela M, Mozes E (2004). Therapeutic vaccines in autoimmunity. Proc Natl Acad Sci USA.

[35] Starr TK, Jameson SC, Hogquist KA (2003). Positive and negative selection of T cells. Annu Rev Immunol.

[36] Szomolanyi-Tsuda E, Welsh RM (1998). T-cell-independent antiviral antibody responses. Curr Opin Immunol.

[37] Tsunoda I, Kuang LQ, Kobayashi-Warren M, Fujinami RS (2005). Central nervous system pathology caused by autoreactive CD8+ T-cell clones following virus infection. J Virol.

[38] Verhoef A, Alexander C, Kay AB, Larche M (2005). T cell epitope immunotherapy induces a CD4+ T cell population with regulatory activity. PLoS Med.

[39] von Herrath MG, Harrison LC (2003). Antigen-induced regulatory T cells in autoimmunity. Nat Rev Immunol.

[40] von Herrath MG, Dockter J, Nerenberg M, Gairin JE, Oldstone MB (1994). Thymic selection and adaptability of cytotoxic T lymphocyte responses in transgenic mice expressing a viral protein in the thymus. J Exp Med.

[41] Walker LS, Abbas AK (2002). The enemy within: keeping self-reactive T cells at bay in the periphery. Nat Rev Immunol.

[42] Yauch RL, Kim BS (1994). A predominant viral epitope recognized by T cells from the periphery and demyelinating lesions of SJL/J mice infected with Theiler’s virus is located within VP1 (233–244). J Immunol.

[43] Yauch RL, Kerekes K, Saujani K, Kim BS (1995). Identification of a major T-cell epitope within VP3 amino acid residues 24 to 37 of Theiler’s virus in demyelination-susceptible SJL/J mice. J Virol.

[44] Yauch RL, Palma JP, Yahikozawa H, Koh CS, Kim BS (1998). Role of individual T-cell epitopes of Theiler’s virus in the pathogenesis of demyelination correlates with the ability to induce a Th1 response. J Virol.

[45] Yewdell JW, Anton LC, Bennink JR (1996). Defective ribosomal products (DRiPs): a major source of antigenic peptides for MHC class I molecules?. J Immunol.

